# Oncolytic Vaccinia Virus Expressing White-Spotted Charr Lectin Regulates Antiviral Response in Tumor Cells and Inhibits Tumor Growth In Vitro and In Vivo

**DOI:** 10.3390/md19060292

**Published:** 2021-05-21

**Authors:** Xue Wang, Ningning Zhou, Tingting Liu, Xiaoyuan Jia, Ting Ye, Kan Chen, Gongchu Li

**Affiliations:** College of Life Sciences and Medicine, Zhejiang Sci-Tech University, Hangzhou 310018, China; wx108@mails.zstu.edu.cn (X.W.); znnzjlg@mails.zstu.edu.cn (N.Z.); liutt@mails.zstu.edu.cn (T.L.); xyjia@zstu.edu.cn (X.J.); yeting@zstu.edu.cn (T.Y.); chenkan@zstu.edu.cn (K.C.)

**Keywords:** white-spotted charr lectin, oncolytic vaccinia virus, interferon, antiviral response

## Abstract

Oncolytic vaccina virus (oncoVV) used for cancer therapy has progressed in recent years. Here, a gene encoding white-spotted charr lectin (WCL) was inserted into an oncoVV vector to form an oncoVV-WCL recombinant virus. OncoVV-WCL induced higher levels of apoptosis and cytotoxicity, and replicated faster than control virus in cancer cells. OncoVV-WCL promoted IRF-3 transcriptional activity to induce higher levels of type I interferons (IFNs) and blocked the IFN-induced antiviral response by inhibiting the activity of IFN-stimulated responsive element (ISRE) and the expression of interferon-stimulated genes (ISGs). The higher levels of viral replication and antitumor activity of oncoVV-WCL were further demonstrated in a mouse xenograft tumor model. Therefore, the engineered oncoVV expressing WCL might provide a new avenue for anticancer gene therapy.

## 1. Introduction

Lectins are a group of proteins that bind carbohydrates reversibly and specifically, and play important roles in various biological processes such as cell division, fertilization, congenital immunity, and cell recognition [1]. Lectins are widely distributed in marine bioresources such as marine cyanobacteria, algae, invertebrate animals and fish. In addition, lectins have shown remarkable abilities in inducing apoptosis and inhibiting angiogenesis, suggesting the future use in treating cancers [2,3]. For example, *Eucheuma serra* agglutinin in red macroalga induced apoptosis in various colon adenocarcinoma, breast cancer and osteosarcoma cells [4,5,6]. *Crenomytilus grayanus* lectin extracted from homonymous bivalve showed globotriaosylceramide (Gb3)-dependent cytotoxicity in breast cancer and Burkitt’s lymphoma cells [7,8]. N-acetyl sugar-binding lectin isolated from *Ibacus novemdentatus* elicited acytotoxic effects on various cancer cells through glycoconjugate interaction [9].

Vaccinia viruses can be used as replicating vectors harboring therapeutic genes to directly lyse tumor cells, or as cancer vaccines to stimulate antitumor immunity [10,11,12]. JX-594, the most famous oncolytic vaccinia virus carrying a human granulocyte-macrophage colony-stimulating factor (GM-CSF) gene with the thymidine kinase (TK) gene deletion, has been advanced to clinical phase III for the treatment of advanced hepatocellular carcinoma (HCC) and clinical phase II trial for renal cell carcinoma [13,14,15,16,17]. In our previous work, genes encoding marine lectins *Tachypleus tridentatus* lectin (TTL) and *Aphrocallistes vastus* lectin (AVL) were inserted into the oncolytic vaccinia virus (oncoVV) vector, forming oncoVV-TTL and oncoVV-AVL recombinant viruses, respectively [18,19]. Compared with the control virus, both oncoVV-TTL and oncoVV-AVL showed faster replication and significant antitumor activities in vitro and in vivo.

Previously, some marine fish lectins have been shown to elicit antitumor effects [2]. In the presented studies, we constructed a new recombinant vaccinia virus oncoVV-WCL, which was generated by inserting a gene encoding white-spotted charr lectin (WCL) into the oncoVV vector. WCL is a type of rhamnose-binding lectin from white-spotted Charr (*Salvelinus leucomaenis*) eggs [20], which has not been previously investigated in oncoVV vectors. We then investigated the antitumor effect of oncoVV-WCL mainly on hepatocellular carcinoma in vitro and in vivo. Lung and cervical cancer cell lines were also used for verification. Simultaneously, the underlying mechanisms of oncoVV-WCL involved in interferon (IFN) production and the IFN-induced antiviral response were elucidated.

## 2. Results

### 2.1. Cytotoxicity of oncoVV-WCL in Cancer Cells

**Figure 1:**
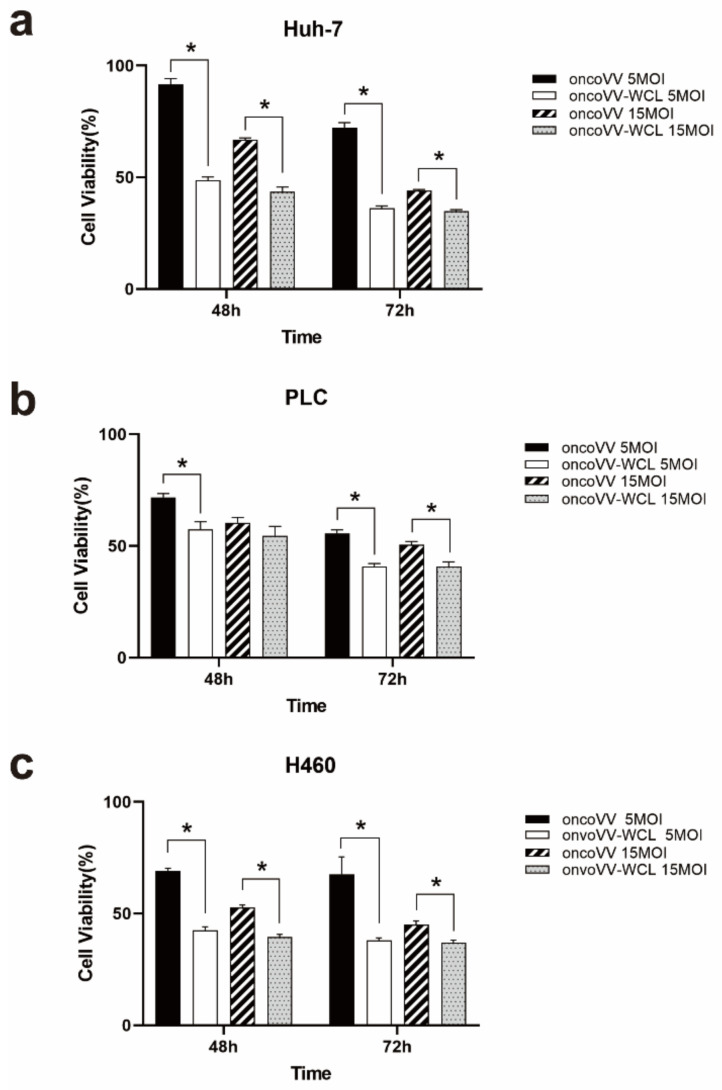
The antiproliferative effect of oncoVV-WCL in tumor cells. Cell viability was measured by MTT assay in Huh-7 cells (**a**), PLC/PRF/5 cells (**b**), H460 cells (**c**). Data are expressed as the mean ± SEM from at least three separate experiments. (* *p* < 0.05).

Hepatocellular carcinoma cells PLC/PRF/5 and Huh-7, as well as lung carcinoma cell H460, were infected with oncoVV or oncoVV-WCL at five multiplicities of infection (MOI) or 15MOI for 48 h and 72 h. An assay of 3-(4,5-dimethylthiazol-2-yl)-5-(3-carboxymethoxy-phenyl)-2-(4-sulfophenyl)-2H-tetrazolium (MTT) was performed to determine cell viability. PBS served as the negative control. The cell viability of the oncoVV-WCL group was decreased compared to the oncoVV group (Figure 1a). The results indicate that exogenous WCL expression enhanced the cytotoxicity of oncoVV in H460, PLC/PRF/5 and Huh-7 cell lines.

### 2.2. Apoptotic Effect of oncoVV-WCL in Huh-7 Cells

**Figure 2:**
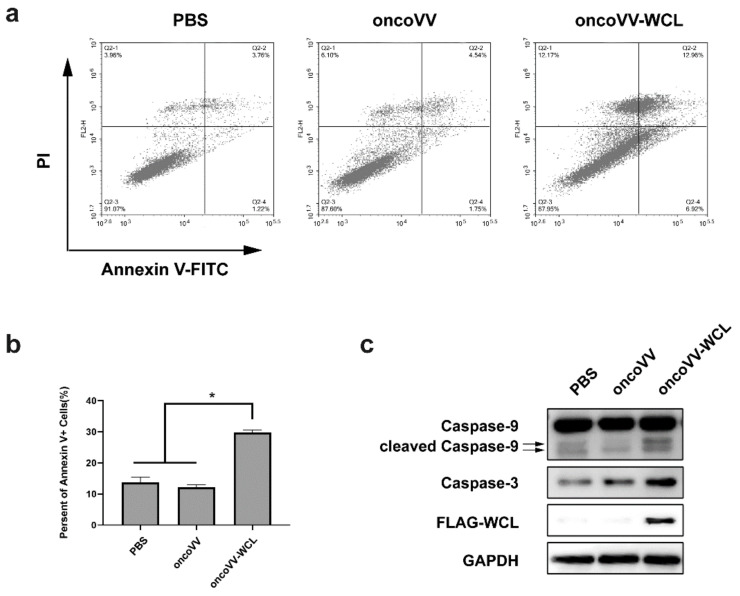
VV-WCL inducing apoptosis in Huh-7 cells. (**a**) Flow cytometric examination. Huh-7 cells were treated with oncoVV or oncoVV-WCL at 5MOI as well as PBS control for 72 h. Cells were stained with Annexin V-FITC and PI followed by analysis under a flow cytometer; (**b**) the percentage of apoptotic cells. Three repeats are represented as mean ± SEM (* *p* < 0.05). (**c**) The expression of Caspase-3, Caspase-9 and FLAG. The expression level was detected by Western blot. GAPDH served as a loading control.

To investigate whether the oncoVV-WCL can induce the apoptosis in cancer cells, Huh-7 cells were treated with oncoVV, oncoVV-WCL and PBS respectively, followed by apoptotic analysis through flow cytometry analysis and Western blot. As shown in Figure 2a,b, compared with PBS and oncoVV, 5 MOI oncoVV-WCL triggered higher apoptosis rates. The infection of oncoVV-WCL increased the expression of caspase-3 and cleaved caspase-9 (Figure 2c), suggesting that the intrinsic apoptotic pathway may play a role in oncoVV-WCL induced cell death.

### 2.3. Replication of Oncolytic Vaccinia Virus Improved by WCL

**Figure 3:**
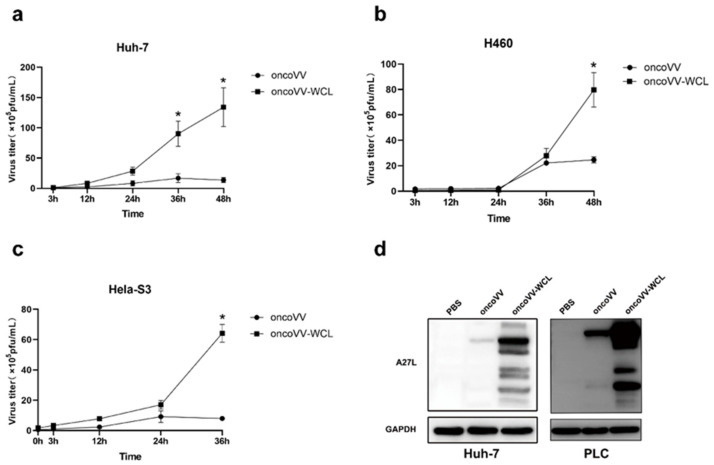
oncoVV-WCL replication in multiple tumor cell lines. The replication of oncoVV-WCL and oncoVV control in Huh-7 cells (**a**), H460 cells (**b**), Hela-S3 cells (**c**). Viral replication was determined by TCID_50_ assay. Data are expressed as the mean ± SEM from at least three separate experiments. (* *p* < 0.05). (**d**) The expression level of A27L was detected by Western blot. PBS served as negative treatment control, GAPDH served as a loading control.

To clarify the underlying mechanism of the antiproliferative effect of oncoVV-WCL, the replication abilities of oncoVV and oncoVV-WCL were tested through the TCID_50_ method in different cancer cells. The results demonstrated that the reproductive number of oncoVV-WCL was much higher than that of oncoVV in Huh-7 cells (Figure 3a), H460 cells (Figure 3b) and Hela-S3 cells (Figure 3c). The expression level of protein A27L, which is located on the surface of the intracellular mature virus [21], was also detected in hepatocellular carcinoma cells Huh-7 and PLC/PRF/5 (Figure 3d). We found the expression level of A27L in oncoVV-WCL treatment group was significantly higher than that in oncoVV treatment group, suggesting that WCL harboring dramatically promotes the replication of vaccinia virus.

### 2.4. Onco VV-WCL Stimulated the Production of Type I IFNs

**Figure 4:**
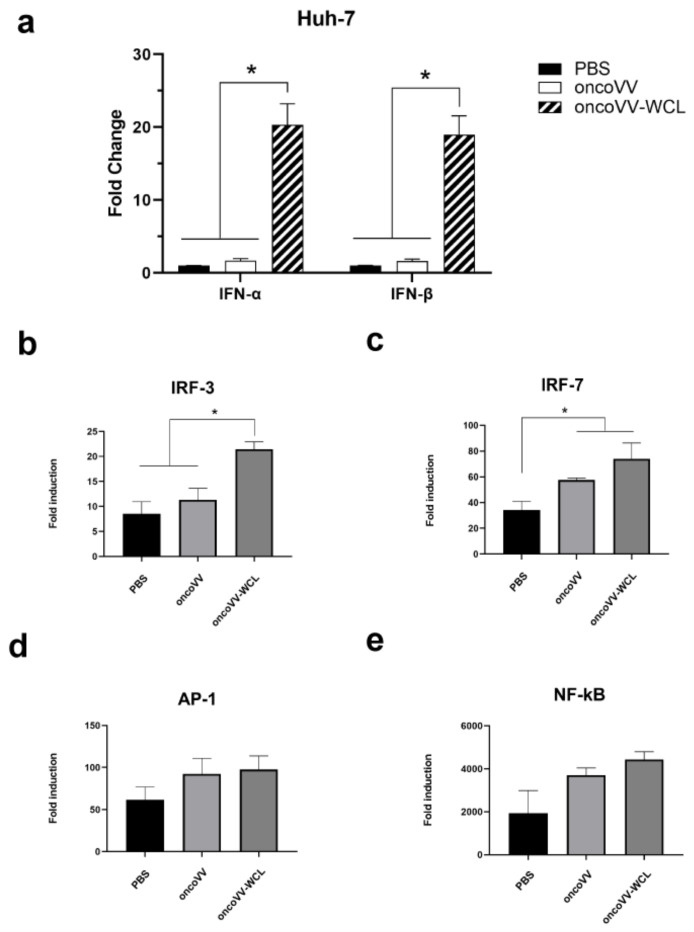
oncoVV-WCL regulates interferon transcription. (**a**) Quantitative RT-PCR evaluations of IFNα/β transcription 48 h after infection of Huh-7 cells with oncoVV or oncoVV-WCL. PBS served as the negative control. The comparative Ct (cycle threshold) method was used to determine RNA expression. Statistically significant differences are presented as * *p* < 0.05. (**b**) oncoVV-WCL activated the transcription activity of IRF-3. Transfection efficiencies were normalized to the pTK-RL luciferase plasmid. Data are expressed as the mean ± SEM from at least three separate experiments. (* *p* < 0.05). Reporter assays of IRF-7 (**c**), AP-1 (**d**), NF-κB (**e**) were detected as described above.

It is well-recognized that IFNs are secreted glycoproteins and are produced from cells in response to virus infection [22]. Vaccinia virus infection activates IFN regulatory factor 3 (IRF3) and IRF7 pathways, and further causes translocation of transcription factors NF-κB and AP-1 into the nucleus, and finally IRF3/7, NF-κB and AP-1 form a complex known as the enhanceosome, driving the transcription of type I IFNs [22]. To investigate the production of interferons by oncoVV-WCL, RNA was extracted from infected Huh-7 cells, and the mRNA levels of IFN-α and IFN-β were evaluated by qRT-PCR. The results demonstrated that the transcription of IFN-α and IFN-β in oncoVV-WCL-treated Huh-7 cells increased dramatically as compared to oncoVV-treated cells (Figure 4a), indicating that WCL enhanced the oncoVV induced production of type I IFNs.

We next preformed the dual-luciferase reporter assay to access the effect of oncoVV-WCL on IRF-3, IRF-7, NF-κB and AP-1 transcription factors. As shown in Figure 4b, the activity of IRF-3 promoters in the oncoVV-WCL treatment group significantly increased compared to that of oncoVV treatment group, while there was no significant difference between oncoVV-WCL and oncoVV treatment groups in IRF-7, AP-1 and NF-κB reporter assays (Figure 4c–e). Therefore, the results suggest that WCL harboring may promote IRF-3 transcription activity to induce the expression of type I IFNs.

### 2.5. OncoVV-WCL Regulated the IFN-Induced Signaling and the Expression of IFN-Stimulated Genes

**Figure 5:**
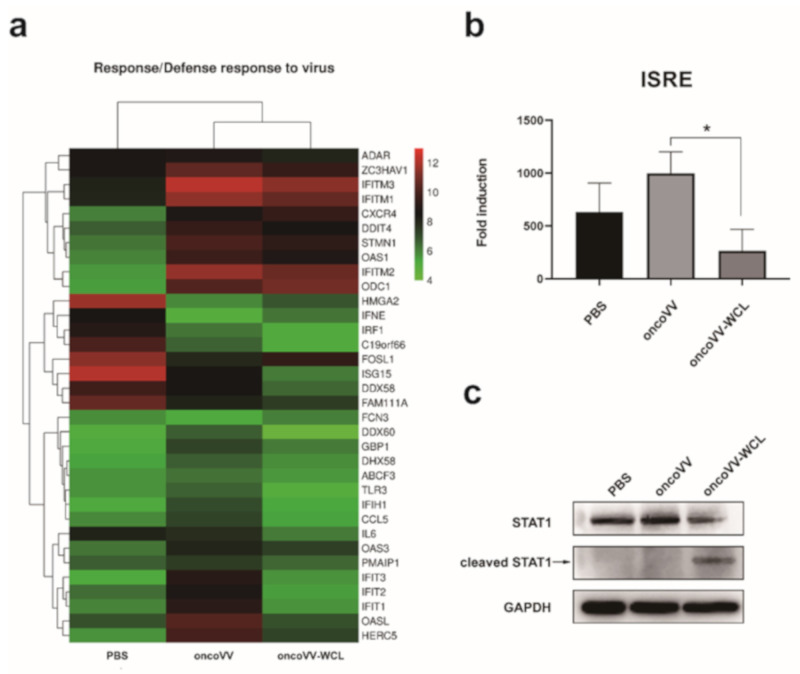
Transcriptomic validation of oncoVV-WCL. (**a**) Heatmap of genes. Huh-7 cells were infected with oncoVV and oncoVV-WCL at 5 MOI for 24 h followed by transcriptomic analysis. The key includes a histogram of the distribution of log_2_ fold change values for all the included genes. (**b**) Activity of ISRE promoter was analyzed through a dual-luciferase reporter assay kit. The transcription activity of ISRE was evaluated by the dual-luciferase reporter assay. Data are represented as mean ± SEM from at least three independent experiments (* *p* < 0.05). (**c**) The expression level of STAT1 was detected by Western blot. GAPDH was used as a loading control.

Because IFNs can induce the transcription of hundreds of interferon-stimulated genes (ISGs) [22], it is reasonable to further profile the effect of oncoVV-WCL on hepatocellular carcinoma cells. Therefore, the gene expressions of Huh-7 cells treated with oncoVV-WCL, oncoVV and PBS were examined through transcriptomic analysis, then a heatmap was created to graphical visualization of differential gene expression. The results showed that 34 genes related to response/defense response to virus were differentially expressed, and among them 25 genes expressions were downregulated by oncoVV-WCL as compared to oncoVV control (Figure 5a). Results suggested that oncoVV-WCL downregulated ISGs expression.

The IFNs induced by virus infection can engage with their cognate receptors and induce Janus-associated kinase (JAK) and the signal transducer and activator of the transcription (STAT) pathway [22,23]. In IFN-α and IFN-β signaling, STAT1 and STAT2 form heterodimers, then associate with IFN regulatory factor 9 (IRF9) to form a trimeric complex, which further translocate into the nucleus to drive the transcription of ISGs associated with the IFN-stimulated responsive element (ISRE) [22,23]. Given that ISGs such as ISG15 and OASL were downregulated (Figure 5a), we postulated that the JAK-STAT signaling cascade pathway might be influenced by the infection of oncoVV-WCL. Therefore, STAT1 expression was tested by Western blot, and the ISRE activity was monitored with the dual-luciferase reporter assay. The results showed that oncoVV-WCL downregulated the activity of ISRE and led to STAT1 cleavage (Figure 5b,c), suggesting that oncoVV-WCL regulated the antiviral response in cancer cells through STAT1 cleavage.

### 2.6. OncoVV-WCL Suppressed Hepatocellular Carcinoma Cell Growth In Vivo

**Figure 6:**
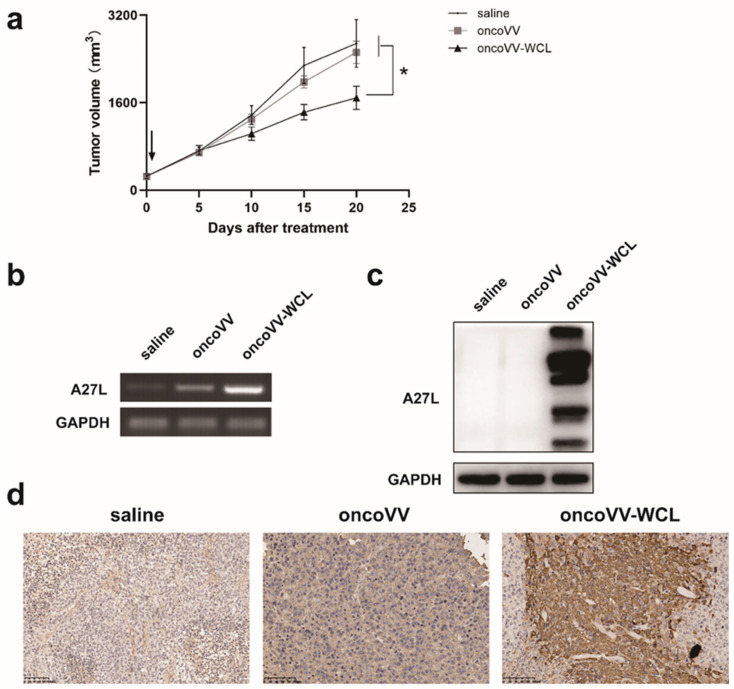
The antitumor effect of oncoVV-WCL in vivo. (**a**) Huh-7 cells were inoculated into the Balb/c nude mice on the back. Tumors were then injected with saline, oncoVV, or oncoVV-WCL, respectively. Data are expressed as the mean ± SEM. (* *p* < 0.05). (**b**) Semiquantitative PCR validated A27L gene expression at the level of RNA. GAPDH served as a loading control. The extracted RNA was reverse transcribed into cDNA and performed this cDNA to semiquantitative PCR followed by agarose gel electrophoresis. GAPDH served as a loading control. (**c**) The expression level of A27L was detected by Western blot. GAPDH served as a loading control. (**d**) The immunohistochemical results of A27L on Huh-7 tumors. Scale bars show 100 μm.

To investigate the antitumor efficacy of oncoVV-WCL in vivo, subcutaneous tumors were established in Balb/c nude mice with Huh-7 cells and then administrated with saline, oncoVV and oncoVV-WCL, respectively. As shown in Figure 6a, the oncoVV-WCL treatment group demonstrated a significant antitumor effect compared with the saline and oncoVV treatment groups. Subsequently, RNA and proteins were extracted from the mice tumors, and semiquantitative PCR and Western blot were performed to test the expression of A27L of virus (Figure 6b,c). Furthermore, the A27L level of oncoVV-WCL and control treatment in mice tumors were also confirmed by immunohistochemistry assay (Figure 6d). The results suggest that the remarkable antitumor effect of oncoVV-WCL may be due to the increasing replication of viruses harboring WCL.

## 3. Discussion

WCL, a family member of lectin, was originally isolated from white-spotted charr eggs in Salmonidae [20]. Since the first report of WCL, there have been few reports studying the function of this protein. Based on our previous study, utilizing an adenovirus or a vaccinia virus carrying a specific lectin manifested a strong antitumor effect. Therefore, investigating the function of an unknown WCL by means of the vaccinia virus intrigued us. To our knowledge, this is the first demonstration of combining a WCL gene with vaccinia virus. This study reports oncoVV harboring WCL can effectively inhibit tumor cell growth both in vitro and in vivo. We further revealed that the recombinant oncoVV-WCL demonstrates high replication in tumor cells and induces type I IFNs production, while inhibiting the expression of ISGs via downregulating the JAK-STAT signaling pathway.

VV used for antitumor therapy has progressed in the past decades [10,24,25,26,27]. It is well recognized that immediately after VV invasion, the innate immunity of the host is activated and then a cascade of signaling pathways involved. To restrict VV replication and spread, the host cells secrete IFNs which launch an immediate large response to attack the virus. Consequently, the viruses express many proteins and evolve multiple strategies to hinder the effect that IFNs produce, including preventing production of IFN, blocking binding of IFNs to IFN receptors, blocking IFN-induced signaling pathways and blocking the antiviral action of ISGs [22]. In our previous reports, oncoVV carrying lectin TTL suppressed the production of IFN-β [18], while in the present study, oncoVV carrying lectin WCL promoted the expression of IFN-α and IFN-β in liver cancer cells. Given that IRF3 NF-κB and AP-1 forming the enhanceosome and binding to IFN-β gene promoter are necessary for the expression of the antiviral IFN-β gene [22,28], our further investigation showed the induction of IFNs might be due to the activation of the key transcription factor IRF-3. Since the VV can impede the effect of IFNs by interfering with the IFN-induced JAK-STAT signaling pathway and the production of ISGs, testing the changes of the JAK-STAT signaling pathway and ISGs is necessary. As shown in the transcriptomic analysis, some ISGs were downregulated by oncoVV-WCL. ISG15 is a ubiquitin-like protein which is induced by viral infection, and IFN-α and -β, and can conjugate to target proteins such as IRF3 [22,29]. IFIT1/2/3 are strongly induced ISGs and function as both sensor and effector molecules of the cellular innate immune system [30,31]. OASL is also a member of ISGs and induced by virus infection rapidly via IFN signaling [32]. The IFIH1 gene encodes a RIG-I-like receptor involved in the sensing of viral RNA [33]. The downregulation of these ISGs might help oncoVV-WCL escape the intracellular antiviral response, thereby achieving a significantly higher level of viral replication in cancer cells both in vitro and in vivo.

We present a novel WCL expressing oncoVV in this study. OncoVV-WCL not only induced type I IFN production, which could elicit antitumor activity but also inhibit IFN-induced ISG production, possibly due to STAT1 cleavage, which helps oncoVV escape elimination. However, oncoVV-WCL only elicited a weak antitumor effect in vivo, as shown. Further investigations into signaling pathways regulating oncoVV-WCL could help to enhance its antitumor activity.

## 4. Materials and Methods

### 4.1. Cell Culture

The human embryonic kidney cell line 293A, hepatocellular carcinoma cell lines Huh-7 and PLC/PRF/5, lung carcinoma cell line H460 and cervical carcinoma cell lines HELA-S3 were provided by the Chinese Academy of Sciences. Cells were cultured in Dulbecco’s Modified Eagle Medium (DMEM)/high glucose medium (Gibco, Thermo Fisher Scientific, Waltham, MA, USA) supplemented with 10% fetal bovine serum (HyClone Laboratories) and 1% Penicillin-Streptomycin on the condition of 37 °C and 5% CO_2_.

### 4.2. Recombination of oncoVV-WCL

The *WCL* gene (GenBank: AB077045.1) was synthesized in Shanghai Generay Biotech Co., Ltd. (Shanghai, China). The oncoVV-WCL recombinant virus was constructed following the steps described previously [18,19]. Briefly, the pCB-Flag-WCL plasmid was constructed by inserting the *WCL* gene into a pCB vector with a thymidine kinase (TK) gene deletion. The 293A cells were infected by wild type vaccinia virus (Western Reverse) for about 3 h and then transfected with pCB-Flag-WCL. The recombinant oncolytic vaccinia viruses were formed 48 h later. Subsequently, the selection of mycophenolic acid, hypoxanthine and dioxopurine were used to select for effective oncoVV-WCL. After three repetitions, purified viruses were obtained. The titers of oncoVV-WCL were determined by TCID_50_ (median tissue culture infective dose).

### 4.3. Western Blot

After cell extraction, equal amounts of proteins were electrophoresed by SDS-PAGE and transferred onto polyvinylidene fluoride membranes (Millipore, Bedford, MA, USA). Then, the membranes were blocked with 5% bovine serum albumin solution at room temperature for 2 h, followed by incubating with primary antibodies at 4 °C overnight. The membranes were washed and incubated with secondary antibodies for 1 h at room temperature. After washing, the membranes were detected using a Tanon 5500 chemiluminescence image system (Tanon Inc., Shanghai, China). The following primary and secondary antibodies were used: Caspase-9 (Abcam, Cambridge, UK, ab202068,1:2000), caspase-3 (Cell Signaling Technology, Danvers, MA, USA, 9662, 1:1000), STAT1 (Santa Cruz, Dallas, TX, USA, sc-346,1:1000), A27L (Abcam, ab35219,1:2000), GAPDH (Cell Signaling Technology, 2118 s, 1:1000), HRP conjugated Goat antiRabbit IgG(H + L) (ABclonal, AS014, 1:5000), HRP conjugated Goat antiMouse IgG(H + L) (ABclonal, Woburn, MA, USA, AS003, 1:5000), and AntiDDDDK-tag pAb (Medical & Biological Laboratories, PM020, 1:1000).

### 4.4. Animal Experiments

Hepatocellular carcinoma tumor-bearing mouse models were built using 6-week-old female Balb/c nude mice (Shanghai Slack Animal Laboratory, Shanghai, China). Mice were housed and raised in accordance with the standards of Animal Care and Use Committee of Zhejiang Sci-Tech University. Huh-7 cells (5 × 10^6^) were subcutaneously inoculated into the back region of mice. When the tumor had grown to reach approximately 250 mm^3^ in size, 1 × 10^7^ plaque-forming units (PFU) oncoVV or oncoVV-WCL, as well as saline, were injected in situ, respectively. Then, tumor volume was recorded and measured every five days. Tumor volume (V) was calculated using the formula: V(mm^3^) = 1/2 (length × width × width) [34,35]. Width was considered as the shorter diameter and length as the longer diameter of the tumor in mm.

### 4.5. Cell Viability Detection and Flow Cytometry Assay

Seeding of 5 × 10^3^/well cells was done in 96-well plates. Cells were infected with oncoVV or oncoVV-WCL at MOIs of 5 and 15. Cell viability was detected by MTT assay. Apoptosis analysis was carried out using the Annexin V-FITC Apoptosis Detection Kit (BD Biosciences, San Jose, CA, USA) according to the manufacturer‘s instructions, and then analyzed by flow cytometer (Accuri C6; BD Biosciences).

### 4.6. Virus Replication Assay

To test the replication of the virus in different cell lines, cells were plated on 24-well plates at a density of 6 × 10^4^/well. Different cells were infected with oncoVV, oncoVV-WCL at and MOI of 5 for 0 h, 3 h, 12 h, 24 h, 36 h and 48 h, respectively. The viral titers were determined through TCID_50_ assay on 293A cells.

### 4.7. Reporter Assay

The dual-luciferase assay kit (GeneCopoeia, Inc., Rockville, MD, USA) was used to perform the reporter assay according to the manufacturer’s instructions. Huh-7 cells were cotransfected with 0.005 μg of pTK-RL plasmid, an internal control encoding Renilla luciferase, and 0.25 μg luciferase reporter plasmids for IRF3, IRF7, NF-κB, AP-1 and ISRE, and then treated with PBS at 5 MOI of oncoVV or oncoVV-WCL for 48 h. After the cells were lysed, the luciferase activities were assessed. The transfection efficiency was normalized to the internal control pTK-RL. All experiments were carried out at least three times.

### 4.8. RNA Extraction and Semi-Quantitative PCR

Total RNA was extracted using Trizol (Invitrogen) reagent according to the manufacturer’s protocol. Then, 2 μg of total RNA was reverse transcribed into complementary DNA (cDNA) using the Sigma Enhanced Avian HS RT–PCR Kit. The products of reverse transcription were used for semiquantitative PCR and detected the expression level of mRNA.

### 4.9. qRT-PCR

IFN-α and IFN-β expression in Huh-7 cells was evaluated by qRT-PCR after infection with oncoVV and oncoVV-WCL for 48 h. After RNA extraction, cDNA was obtained. The qRT-PCR reaction was carried out using a MYiQ single-color real-time PCR detection system. The reaction volume of 20μL contained 1 uL cDNA,320 nM each primer and 10 uL iQSYBR green supermix. The reaction performed at 95 °C for 10 min, then 40 PCR cycles of denaturation at 95 °C for 10 s and annealing at 60 °C for 1 min. Targeted gene expression was normalized to GAPDH messenger RNA. Experiments were repeated three times.

### 4.10. Transcriptomic Analysis

Huh-7 cells were infected with oncoVV and oncoVV-WCL at five MOI for 24 h. PBS treatment served as negative control. The cells were harvested in TRIzol reagent (Invitrogen, Waltham, MA, USA) and submitted to Shanghai Baygene Biotechnologies Co., Ltd. (Shanghai, China) for analysis and profiling the differentially expressed gene. Briefly, the total RNA was extracted using Trizol and a gene chip assay was performed using Clariom D human Affymetrix gene chip (Affymetrix, Shanghai, China), GeneChip WT PLUS Reagent Kit (Affymetrix, 902923), and GeneChip Hybridization, Wash and Stain Kit (Affymetrix, 900720). 

### 4.11. Immunohistochemistry

On day 10 post-injection of viruses, the tumors were harvested for immunohistochemistry (IHC) analysis. The IHC was performed by HaoKe Biotechnology Co. Ltd. (Hangzhou, China). Briefly, after fixation, the tumors were embedded in paraffin, deparaffinized with xylene, and rehydrated with graded alcohol washes and H_2_O_2_. Antigen retrieval was performed with EDTA retrieval buffer. The endogenous peroxidase of samples was blocked followed by protein block with 3% BSA. Subsequently, slides were incubated with primary AntiA27L rabbit antibody at 4 °C overnight, washed with PBS and incubated with secondary HRP conjugated goat antiRabbit IgG (Abcam, ab97051, 1:200) for 50 min at room temperature then stained with DAB solution, counterstained with hematoxylin, dehydrated and mounted for examination. The PBS treatment group served as negative control.

### 4.12. Statistical Analysis

Statistical analysis was determined by student’s *t*-test. Significance was considered as *p* < 0.05.

## 5. Conclusions

Our study showed the newly constructed oncoVV-WCL induced significant apoptotic cell death in hepatocellular carcinoma cells and lung carcinoma cells. The animal experiments verified the in vivo antitumor activity of oncoVV-WCL. WCL harboring promoted vaccinia virus replication and production of type I IFNs via upregulation of IRF-3 activity in hepatocellular carcinoma cells. The underlying mechanisms of antitumor activity of oncoVV-WCL might be due to the inhibition of the transcriptional activity of ISRE and the expression of ISGs thereby resulted in the inhibition of the antiviral response and the promotion of viral replication in cancer cells. Our findings may provide insights into oncolytic viral therapies armed with WCL.

## Data Availability

Data are contained within the article.
